# A New Variant of Avian Encephalomyelitis Virus Associated with Neurologic Signs in Turkey Poults

**DOI:** 10.3390/pathogens13090758

**Published:** 2024-09-04

**Authors:** Gun Temeeyasen, Tamer Sharafeldin, Saad Gharaibeh, Nader M. Sobhy, Robert E. Porter, Sunil K. Mor

**Affiliations:** 1 Animal Disease Research and Diagnostic Laboratory, Department of Veterinary and Biomedical Sciences, South Dakota State University, Brookings, SD 57007, USA; gun.temeeyasen@sdstate.edu (G.T.); tamer.sharafeldin@sdstate.edu (T.S.); 2 Department of Pathology, Faculty of Veterinary Medicine, Zagazig University, Zagazig 44511, Sharkia, Egypt; 3 Veterinary Diagnostic Laboratory, Department of Veterinary Population Medicine, University of Minnesota, Saint Paul, MN 55455, USA; sgharaib@umn.edu (S.G.); nyaacoob@umn.edu (N.M.S.); porte349@umn.edu (R.E.P.); 4 Department of Animal Medicine, Infectious Diseases, Faculty of Veterinary Medicine, Zagazig University, Zagazig 44511, Sharkia, Egypt

**Keywords:** avian encephalomyelitis, tremovirus A1, variant, phylogeny

## Abstract

Avian encephalomyelitis (AE) is a disease caused by the avian encephalomyelitis virus (AEV) of the genus Tremovirus in the family Picornaviridae. Recently, cases of turkey poults showing neurological signs were submitted to the veterinary diagnostic laboratories at South Dakota State University and the University of Minnesota. The affected birds were showing nervous neurological signs such as tremors, inability to stand, torticollis, and wing drop. Clinical signs were observed by 3 weeks of age. Necropsy of birds revealed no significant gross lesions in the internal organs, including the brain. There was no significant bacterial growth in the brains. Microscopic examination of various sections of the brain revealed multifocal lymphocplasmacytic perivascular cuffs in the cerebellum and cerebral cortex. The brain samples were processed for detection and whole genome sequencing by next-generation sequencing. Three full-length polyprotein sequences (6405 nt) of AEV were assembled. All three sequences shared 99.9–100% nucleotide and 100% amino acid identities with each other. Only 77.7–78.5% of nucleotide and 90.3–92.5% of amino acid identities with AEV field strains and vaccine sequences were available in GenBank. This indicates that a new divergent variant of AEV is circulating in the field and causing AE outbreaks in the Midwest region.

## 1. Introduction

Avian encephalomyelitis (AE) is a disease caused by avian encephalomyelitis virus (AEV), a positive-sense, single-stranded RNA genome of genus *Tremovirus* in the family *Picornaviridae* [1]. The genome of AEV consists of only a single open reading frame (ORF) that encodes a large polyprotein of 2143 amino acids. Proteolytic cleavage by viral protease converts this polyprotein into mature proteins [2]. The AEV has a wide susceptibility range in multiple avian species, including chickens, turkeys, pheasants, and quails. This virus causes decreased egg production in layers and neurological disorders such as tremors, ataxia, head tilt, and paralysis in young birds. AEV is a worldwide disease that was first identified in the United States in 1930. AEV infection is generally confirmed either by detection of the virus, using PCR or virus isolation [3,4,5,6], or serological tests associated with the observed clinical signs [7,8,9,10,11]. Although there is no cure for this disease, the widely used, commercially available vaccine seems to be effective in controlling its spread [12]. However, the safety of the vaccine is still controversial [5].

This current report describes the infection of several U.S. turkey flocks with novel variant AEV recovered from the brains of affected turkey poults, in which the poults exhibited neurological signs. 

## 2. Materials and Methods

### 2.1. Clinical Cases

In 2022, three White Holland turkey farms in the U.S. experienced an outbreak of neurological disease in which three-week-old poults were ataxic with dropped wings progressing to tremors, inability to stand, torticollis, and paddling on their sides. The cases were submitted to the South Dakota State University, the Animal Disease Research and Diagnostic Laboratory (SDSU-ADRDL), and the University of Minnesota, Veterinary Diagnostic Laboratory (UMN-VDL). A complete necropsy was performed on each case. Brain swabs were collected for bacterial culture to rule out bacterial encephalitis. 

### 2.2. Histopathology

A set of tissues from each case were collected in neutral buffered formalin (Fisherbrand, Pittsburgh, PA, USA) and processed for histopathology. Briefly, tissues were fixed overnight in 10% neutral buffered formalin, trimmed, embedded in paraffin blocks, cut, mounted on glass slides, and stained with hematoxylin (Epredia, Kalamazoo, MI, USA) and eosin (Leica, Richmond, IL, USA). 

### 2.3. Sample Processing and RNA Extraction

Pooled brain tissues from the same case were diluted 1:10 with phosphate buffer saline (PBS, Corning, Manassas, VA, USA) before tissue homogenization by stomacher. Pooled brain homogenates were centrifuged at 15,000× *g* for 1 min and supernatants were collected for RNA extraction.

A cocktail of DNase and RNase, including Ambion™ DNase I, Turbo DNase, RNase cocktail, RNase I (Invitrogen, Vilnius, Lithuania), Baseline ZERO DNase (LGC Biosearch Technologies, Middleton, WI, USA), and Pierce™ Universal Nuclease for Cell Lysis (Thermo Scientific, Vilnius, Lithuania), was used for the nuclease treatment to digest the unprotected nucleic acids in supernatants from brain homogenates. RNA was extracted using the QIAamp viral RNA mini kit (Qiagen, Hilden, Germany) according to the manufacturer’s instruction, except for the lysis buffer AVL which did not contain carrier RNA.

### 2.4. Metagenomic Sequencing

Reverse transcription and second strand synthesis were performed using the SuperScript III First Strand Synthesis System (Invitrogen, Carlsbad, CA, USA) and Sequenase Version 2.0 DNA Polymerase (Applied Biosystems, Vilnius, Lithuania) with barcoded random hexamers FR26RV-N [13]. Complimentary DNAs (cDNAs) were then amplified by using TaKaRa rTaq with barcode primers FR20RV [13]. One nanogram of the resulting amplicons from each sample was used for library preparation by using the Nextera XT library preparation kit (Illumina, San Diego, CA, USA) and was sequenced on an Illumina MiSeq machine with 300 cycles. The raw fastq files were processed for de novo assembly by using the CLC Genomics Workbench version 22.0.2 (Qiagen, Aarhus, Denmark) and the contigs were identified by BLASTX using the functional analysis module of OmicsBox (BioBam, Valencia, Spain).

### 2.5. Whole Genome Sequence Analysis

The obtained sequences were aligned with the published sequences from GenBank (Supplementary Data S1) by using the Clustal W program [14]. Phylogenetic trees were constructed based on the nucleotide sequence of the full-length polyprotein, P1 region, VP1, and VP2 genes by using the Maximum Likelihood method and Tamura–Nei model [15] in MEGA X. The bootstrap values were determined by 1000 replicates. The full-length polyprotein sequences of AEV discovered in these three cases were deposited in GenBank (accession numbers OR105999-OR106001). The newly characterized strains were compared with the vaccinal reference strain “Calnek” (GenBank accession no. NC003990).

## 3. Results

### 3.1. Macroscopic and Microscopic Lesions

Gross necropsy and histopathological findings in the three field cases were similar. There were no significant macroscopic lesions in any organ. No remarkable gross lesions were observed in the brains and no bacterial growth was recovered from the brains. 

**Figure 1:**
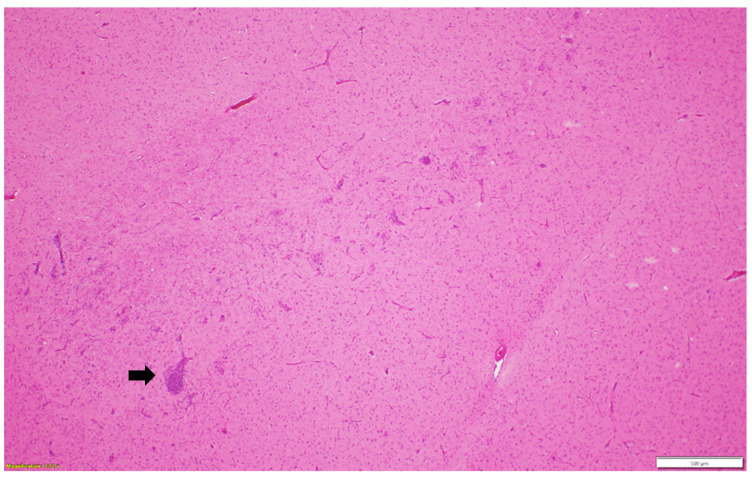
Multifocal lymphoplasmacytic encephalitis and perivascular cuffing (black arrow) in the cerebral white matter. Paraffin-embedded tissue was stained with hematoxylin and eosin.

**Figure 2:**
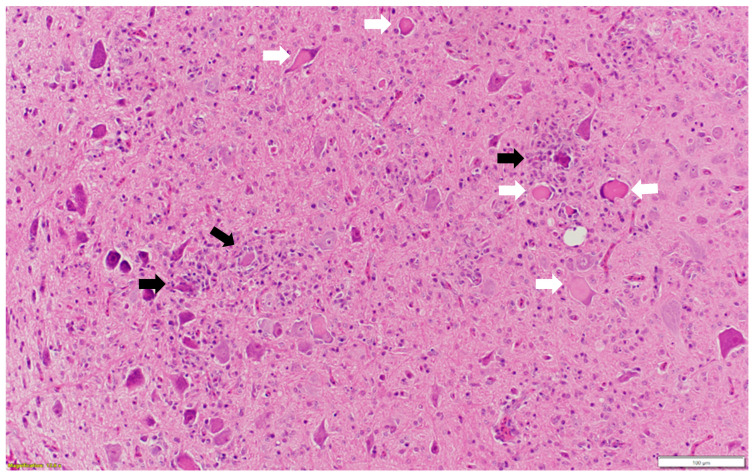
Multiple neurons with central chromatolysis (white arrow: pathognomonic for AEV) and inflammatory cells around degenerated neurons (black arrow: satellitosis and neuronophagia). Paraffin-embedded tissue was stained with hematoxylin and eosin.

**Figure 3:**
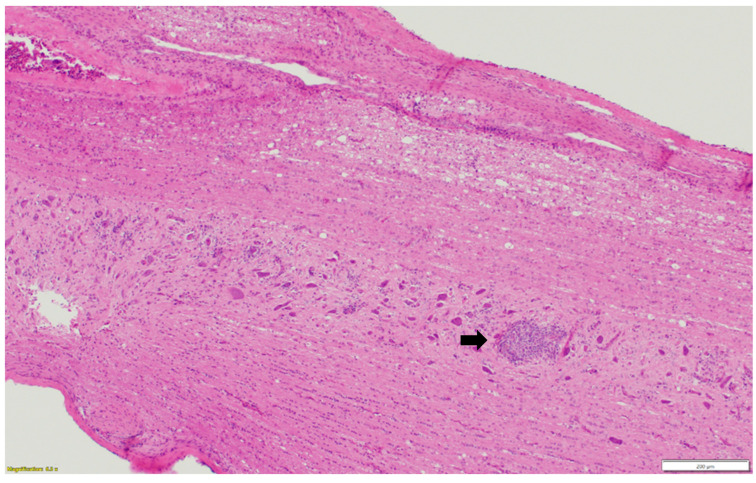
Multifocal lymphoplasmacytic infiltration (black arrow) in the gray matter of spinal cord. Paraffin-embedded tissue was stained with hematoxylin and eosin.

**Figure 4:**
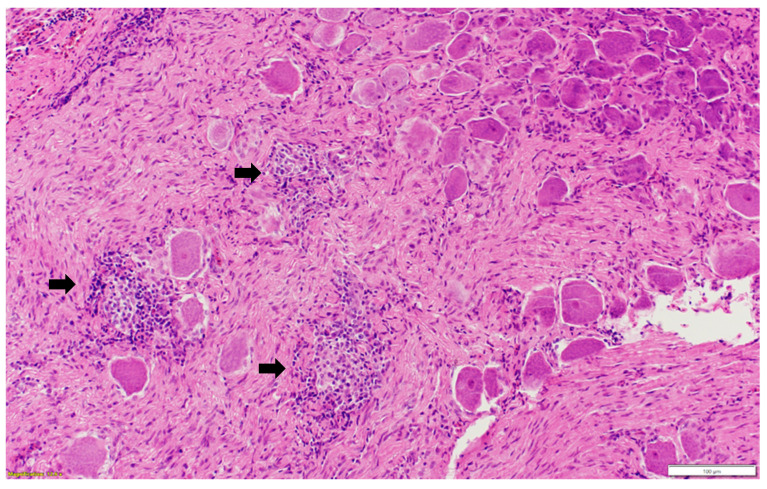
Multifocal inflammatory cellular infiltration (black arrow) among the ganglionic neurons (ganglioneuritis) in the pelvic ganglion. Paraffin-embedded tissue was stained with hematoxylin and eosin.

Microscopic examination of different brain, spinal cord, peripheral nerves, and ganglions sections revealed multifocal areas of lymphoplasmacytic and histiocytic infiltrates, indicating encephalitis (Figure 1) in the cerebral white matter, thalamus, hippocampus, and medulla oblongata close the ventricular space. The inflammatory cells around the vessels (perivascular cuffing) as well as around the neurons (satellitosis and neuronophagia) resulted in several neuronal degeneration and central chromatolysis (Figure 2). The gray matter in the spinal cord had multifocal lymphoplasmacytic infiltrates, indicating myelitis (Figure 3). In addition, there was mild to moderate multifocal ganglioneuritis in the pelvis ganglions (Figure 4).

### 3.2. Whole Genome Sequence Analysis

Three full-length polyprotein sequences (6405 nt) were assembled de novo from three cases. The nucleotide and amino acid identities among full-length polyprotein of these three sequences were 99.9% and 100%, respectively; meanwhile, the nucleotide and amino acid identities between these three sequences with published sequences in GenBank (Supplementary Data S1) were 77.7–78.5% and 90.3–92.5%, respectively. When considering only the P1 region which encoded the structural protein of AEV, the nucleotide and amino acid identities among these three sequences were 99.9–100% and 100%, respectively; meanwhile, the nucleotide identities between these and the published sequences in GenBank were 78.3–79.7% and 93.3–95.2%, respectively (Table 1). In addition, the P2 and P3 regions (which encoded non-structural proteins and RNA polymerase, respectively) of these three sequences also had low nucleotide and amino acid identities with published sequences in GenBank at 77.0–78.0% and 88.6–91.0%, respectively (Table 1). This indicated a genetic distinction between these three novel AEV sequences with the previous AEV sequences that were retrieved from GenBank.

**Figure 5:**
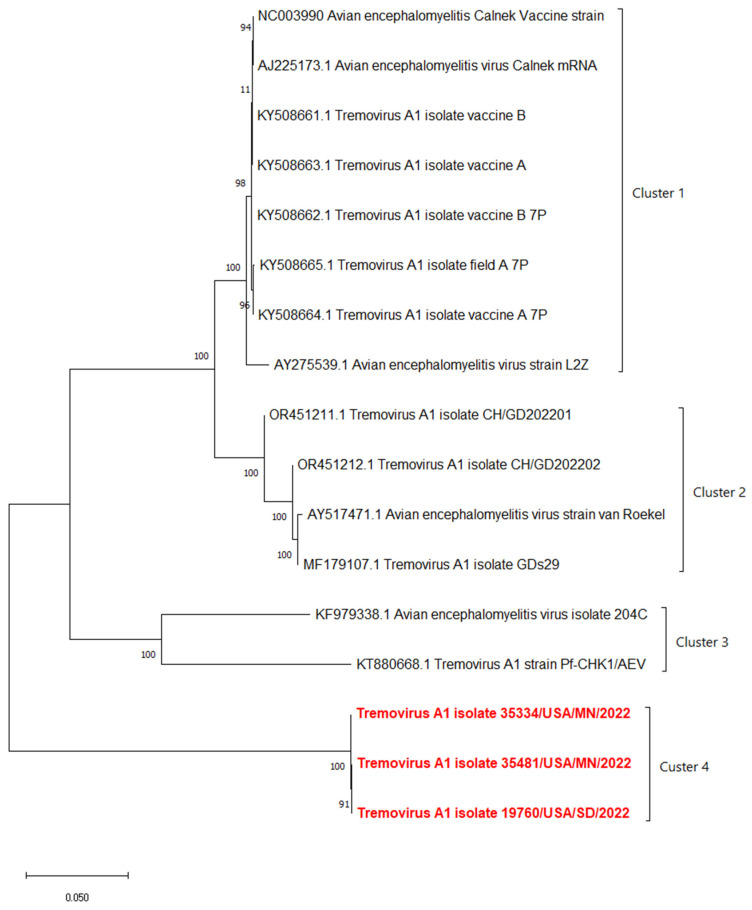
Phylogenetic tree based on nucleotide sequence of full-length polyprotein of AEVs. The evolutionary history was inferred by using the Maximum Likelihood method and Tamura–Nei model with 1000 replicates bootstrap. Sequences determined in this work are highlighted in red.

**Figure 6:**
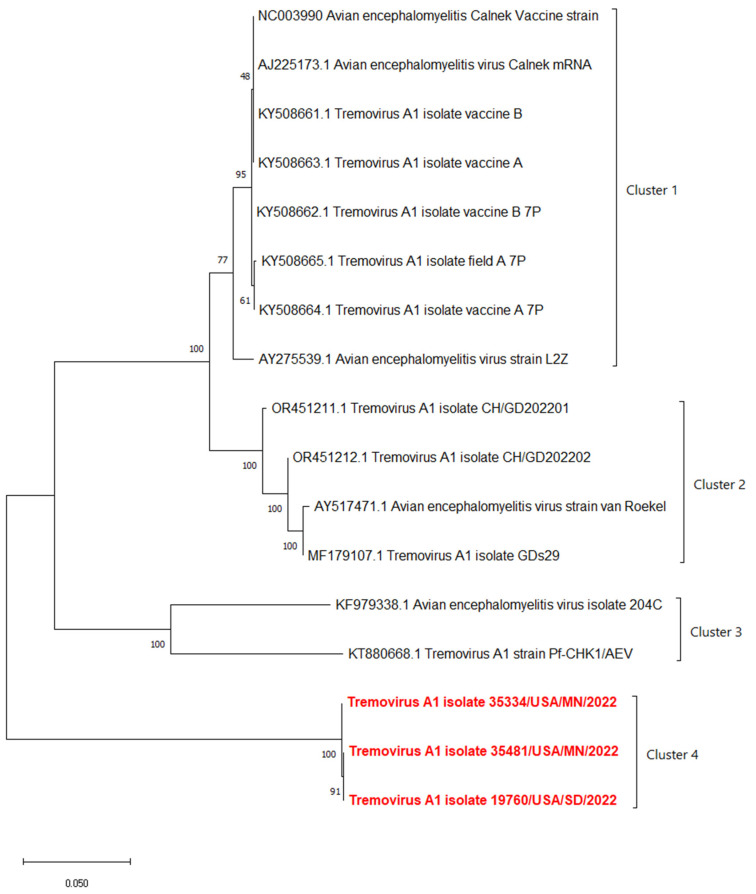
Phylogenetic tree based on nucleotide sequence of P1 region protein of AEVs. The evolutionary history was inferred by using the Maximum Likelihood method and Tamura–Nei model with 1000 replicates bootstrap. Sequences determined in this work are highlighted in red.

**Figure 7:**
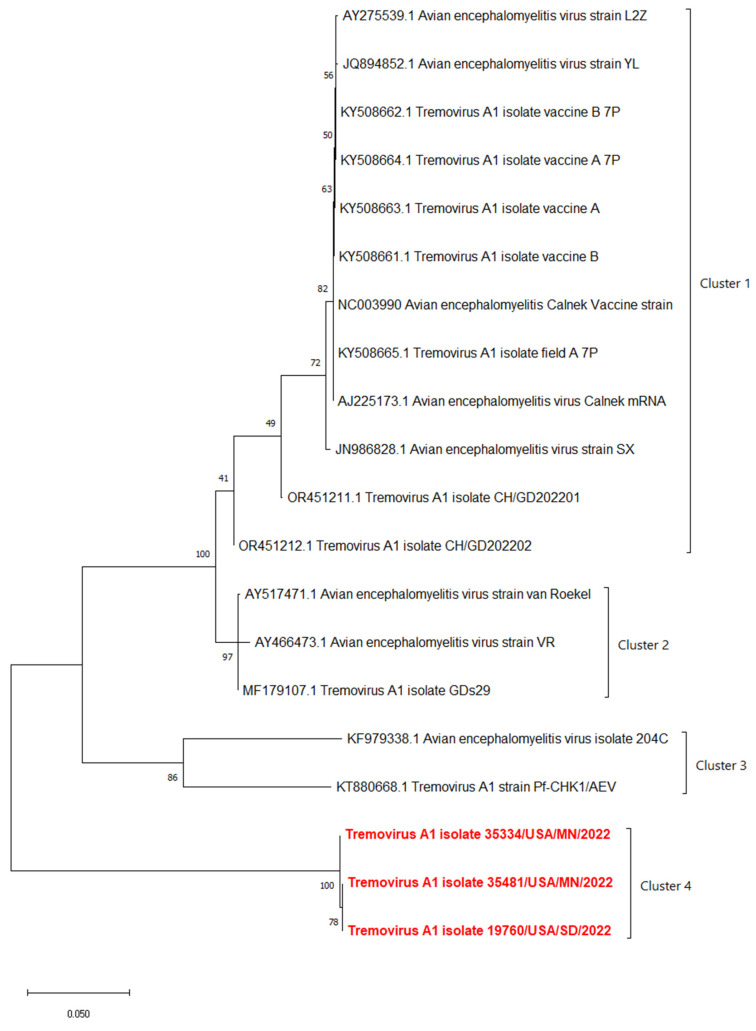
Phylogenetic tree based on nucleotide sequence of VP1 protein of AEVs. The evolutionary history was inferred by using the Maximum Likelihood method and Tamura–Nei model with 1000 replicates bootstrap. Sequences determined in this work are highlighted in red.

**Figure 8:**
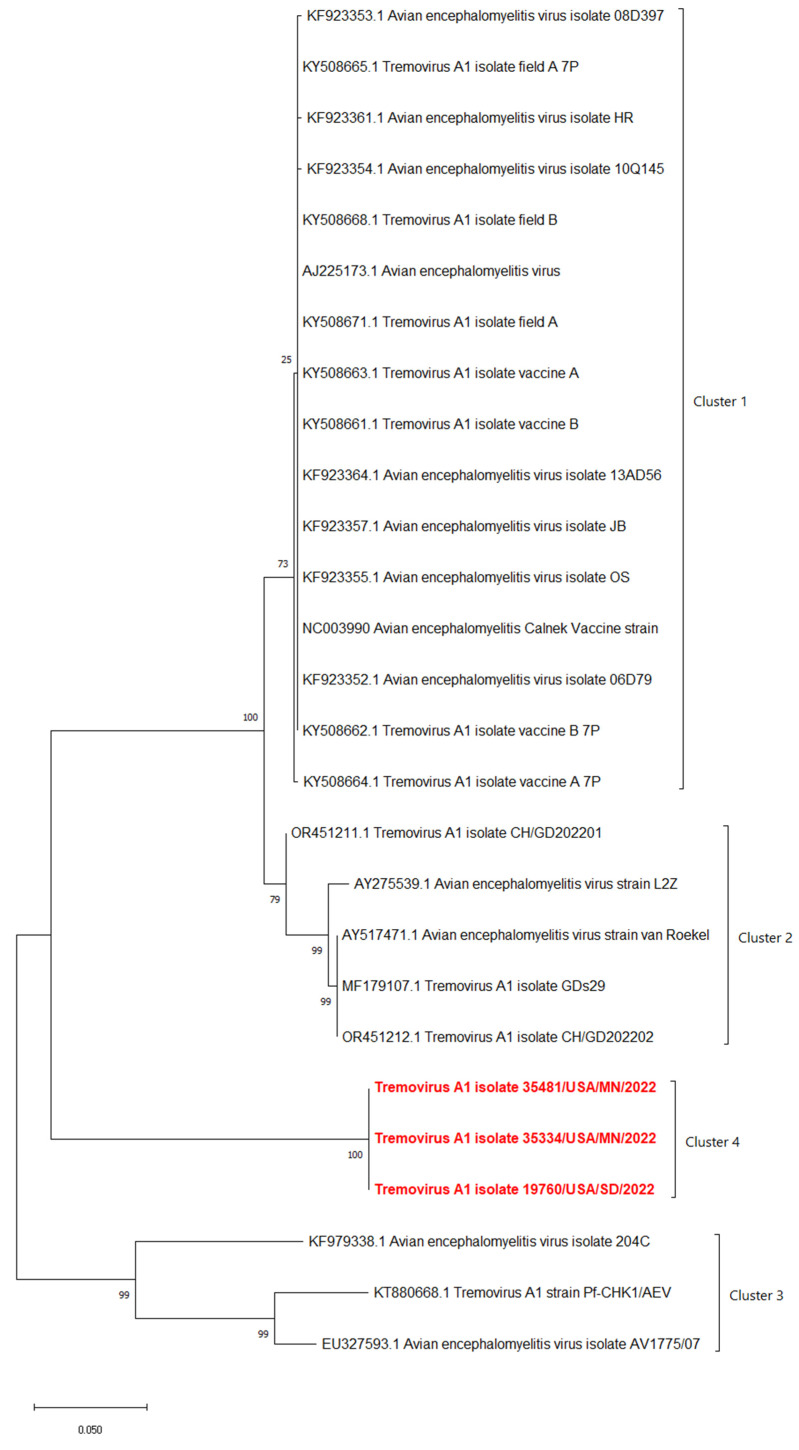
Phylogenetic tree based on nucleotide sequence of VP2 protein of AEVs. The evolutionary history was inferred by using the Maximum Likelihood method and Tamura–Nei model with 1000 replicates bootstrap. Sequences determined in this work are highlighted in red.

**Figure 9:**
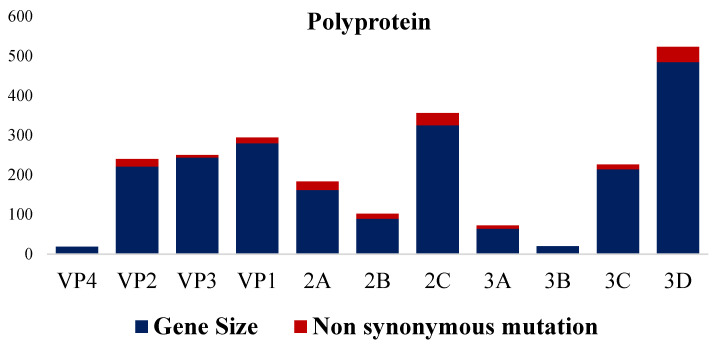
Percentage of non-synonymous aa mutations in comparison with NC003990 vaccinal strain.

The phylogenetic analyses based on the full-length polyprotein, P1 region, and VP1 showed the same pattern in which these three novel sequences were grouped together into a unique cluster separated from previous sequences (Figure 5, Figure 6 and Figure 7). Although these three sequences were grouped in between the main cluster, with embryo-adapted van Roekel (AY517471) strain [16], and another unique cluster that included the isolate from Hong Kong (KF979338), Hungary (KT880668), and UK (EU327593) when the VP2 gene was used for phylogenetic analysis (Figure 8), the nucleotide and amino acid identities of novel sequences with the sequences in the main group (79.4–81.0% and 95.5–97.7%, respectively) were still lower than the nucleotide and amino acid identities between the sequences in the main group and another unique group (80.3–82.5% and 96.1–100%, respectively). These results also indicated that the novel AEVs discovered in this study were genetically distinct from the previous AEVs in GenBank. The comparison with the vaccinal reference strain revealed that non-synonymous substitution was present along the genome, including structural and non-structural proteins with different percentages (Figure 9).

Analysis of the deduced amino acids revealed 126 unique amino acids scattered all around the genomes of the three sequences in this study when compared with the published sequences in GenBank. Interestingly, the majority of these unique amino acids (93 out of 126 aa) were located in P2 (52 aa) and P3 (41 aa); meanwhile, the P1 region, which encoded structural protein, has 33 unique amino acids when compared to the published sequences in GenBank. However, VP1 (18 aa) has the highest unique aa in the P1 region followed by VP3 (10 aa) and VP2 (5 aa), while VP4 has none. In the P2 region, nonstructural protein 2C has the highest unique aa (26 aa) followed by 2B (17 aa) and 2B (9 aa). Lastly, the distribution of unique aa in the P3 region was 3D (24 aa), 3C (11 aa), and 3A (6 aa), while none were found in 3B (Supplementary Data S2).

## 4. Discussion

Detection of AEV in the brains of 2–3-week-old turkey poults with evident pathognomonic microscopic lesions raised the question of whether this is a new variant of AEV. From a large ORF that encodes only a single polyprotein of AEV, the P1 region encodes a structural protein that is commonly used as a molecular detection marker. In particular, P1 contains the coat proteins VP1 and VP2. VP1 is considered a host-protective immunogen that induces neutralizing antibodies while VP2 is the protein that differentiates the pathological types of AEV into natural wild viruses with intestinal tropism versus embryo-adapted neurotropic strains [16,17]. Non-structure proteins, P2 and P3, are necessary for starting viral RNA synthesis and polyprotein processing in the picornavirus life cycle. Hence, mutated 3C can lead to a direct effect on viral pathogenesis and pathogen–host interaction [18]. The single-stranded RNA viruses were reported to have the fastest rate of non-structure protein mutation to increase virus fitness for adapting to different host and host conditions [19]. Mutation in the detected AEV RdRp can indicate a novel viral adaptation process [20].

In this study, the genomic and phylogenetic analyses of full-length polyprotein, P1 region, VP1, and VP2 proteins demonstrated that all three AEVs in this study had unique characteristics when compared to previously published AEV sequences. In fact, all three AEV sequences from field cases in this study were grouped into a separate cluster regardless of what protein was used in the analysis. The deduced amino acid analysis also revealed the unique amino acids scattered all around their genomes. Although the majority of these unique aa were not in the P1 region (which encoded antigenic structural proteins), most of the unique aa were located in the VP1 gene, which might indicate the virus mutated through positive pressure. However, the majority of the unique aa found in the P2 and P3 regions might also indicate a negative mutation as well. These findings indicate that all three AEVs in this study are novel variants that are first described here.

Historically, AEV sequences from all over the world are highly conserved, and their corresponding antigenic properties represent only one serotype of AEV. However, the novel AEVs from this study shared lower nucleotide and amino acid identities than the previous sequences, which might affect the serological reactivity. Therefore, the cross-reaction of antibodies against previous AEV isolates with these novel AEVs would be needed to elucidate whether the novel AEVs are still the same serotype as previous AEVs. Moreover, variation along the AEV genome can obstruct accurate molecular detection of AEV on farms as misdiagnosis with known primers can allow infections to persist for a longer time, increasing the chance for the accumulation of more mutations and complicating the endemicity of the infection [21].

Genetic mutation can phenotypically be expressed by alteration of virulence and pathogenicity of vaccinal strain, which is common practice in picornaviruses [22]. In a 2016 report, vaccine-associated AE outbreaks occurred in Leghorn layer pullets [5]. In that report, neurological signs were observed within 2 weeks of the flock receiving a wing-web injection of the combined product of fowl pox and the AE vaccine. Previous studies demonstrated that two point mutations in the VP2 (nt position 626) and VP3 (nt position 914) induce a change in tissue tropism of AE; an embryo-adapted neurotropic phenotype would cause the clinical signs only after intracerebral inoculation or subcutaneous injection [17]. Although none of the sequences from novel AEVs in this study showed the embryo-adapted genotype, leading the possibility of vaccination-induced outbreak to be low, the cause of these recent outbreaks needs to be further investigated. 

## 5. Conclusions

In conclusion, a novel variant AEV was discovered in the brains of turkey poults that had neurological signs. Genetic and phylogenetic analyses indicated that this novel variant had genetical distinct from published AEVs in the GenBank. This is the first report of a novel divergent AEV outbreak in turkey pouts in the USA.

## Figures and Tables

**Table 1:** Nucleotide and amino acid identities of novel AEVs and published AEVs in GenBank.

Genes	Between Novels AEVs Nucleotide (Amino Acid)	With Previous AEVs Nucleotide (Amino Acid)
Full-length polyprotein	99.9% (100%)	77.7–78.5% (90.3–92.5%)
P1 region	99.9–100% (100%)	78.3–79.7% (93.3–95.2%)
- VP1	99.8–100% (100%)	77.2–79.7% (92.5–93.3%)
- VP2	100% (100%)	79.4–81.0% (95.5–97.7%)
- VP3	99.8–100% (100%)	77.1–79.4% (91.4–95.9%)
P2-P3 regions	99.9–100% (100%)	77.0–78.0% (88.6–91.0%)

## Data Availability

The full-length polyprotein sequences of AEV discovered in this study were deposited in GenBank under accession numbers OR105999-OR106001.

## Supplementary Materials

The following supporting information can be downloaded at: https://www.mdpi.com/article/10.3390/pathogens13090758/s1, Supplementary Data S1: List of published AEV sequences from GenBank that were used in this study.; Supplementary Data S2: Deduced amino acid analysis of these 3 novel variants found in this study with the published sequences from GenBank. The red box indicated the unique amino acids that were found only in these 3 novel AEV from this study.
