# Supplementary material for: A New Variant of Avian Encephalomyelitis Virus Associated with Neurologic Signs in Turkey Poults
Source: Pathogens. 2024 Sep 4;13(9):758. doi: 10.3390/pathogens13090758 (PMC11434790; doi:10.3390/pathogens13090758)
Supplement: Supplementary file 1 [file pathogens-13-00758-s001.zip › pathogens-3150323-supplementary.pdf]

Supplementary data S1. List of published AEV sequences from GenBank that were used in this study.

Accession number	Strain	Gene
NC003990	Calnek vaccine strain	Full-length polyprotein
AJ225173	Calnek mRNA	Full-length polyprotein
KY508661	Vaccine B	Full-length polyprotein
KU508663	Vaccine A	Full-length polyprotein
KY508662	Vaccine B 7P	Full-length polyprotein
KY508665	Field A 7P	Full-length polyprotein
KY508664	Vaccine A 7P	Full-length polyprotein
AY275539	L2Z	Full-length polyprotein
OR451211	CH/GD202201	Full-length polyprotein
OR451212	CH/GD202202	Full-length polyprotein
AY517471	Van Reokel	Full-length polyprotein
MF179107	GDs29	Full-length polyprotein
KF979338	204C	Full-length polyprotein
KT880668	Pf-CHK1/AEV	Full-length polyprotein
JQ894852	YL	VP1
JN986828	SX	VP1
AY466473	VR	VP1
KF923353	08D397	VP2
KF923361	HR	VP2
KF923354	10Q145	VP2
KY508668	Field B	VP2
KY508671	Field A	VP2
KF923364	13AD56	VP2
KF923357	JB	VP2
KF923355	OS	VP2
KF923352	06D79	VP2
EU327593	AV1775/07	VP2

Supplementary data S2. Deduced amino acid analysis of these 3 novel variants found in this study with the published sequences from GenBank. The red box indicated the unique amino acids that were found only in these 3 novel AEV from this study.

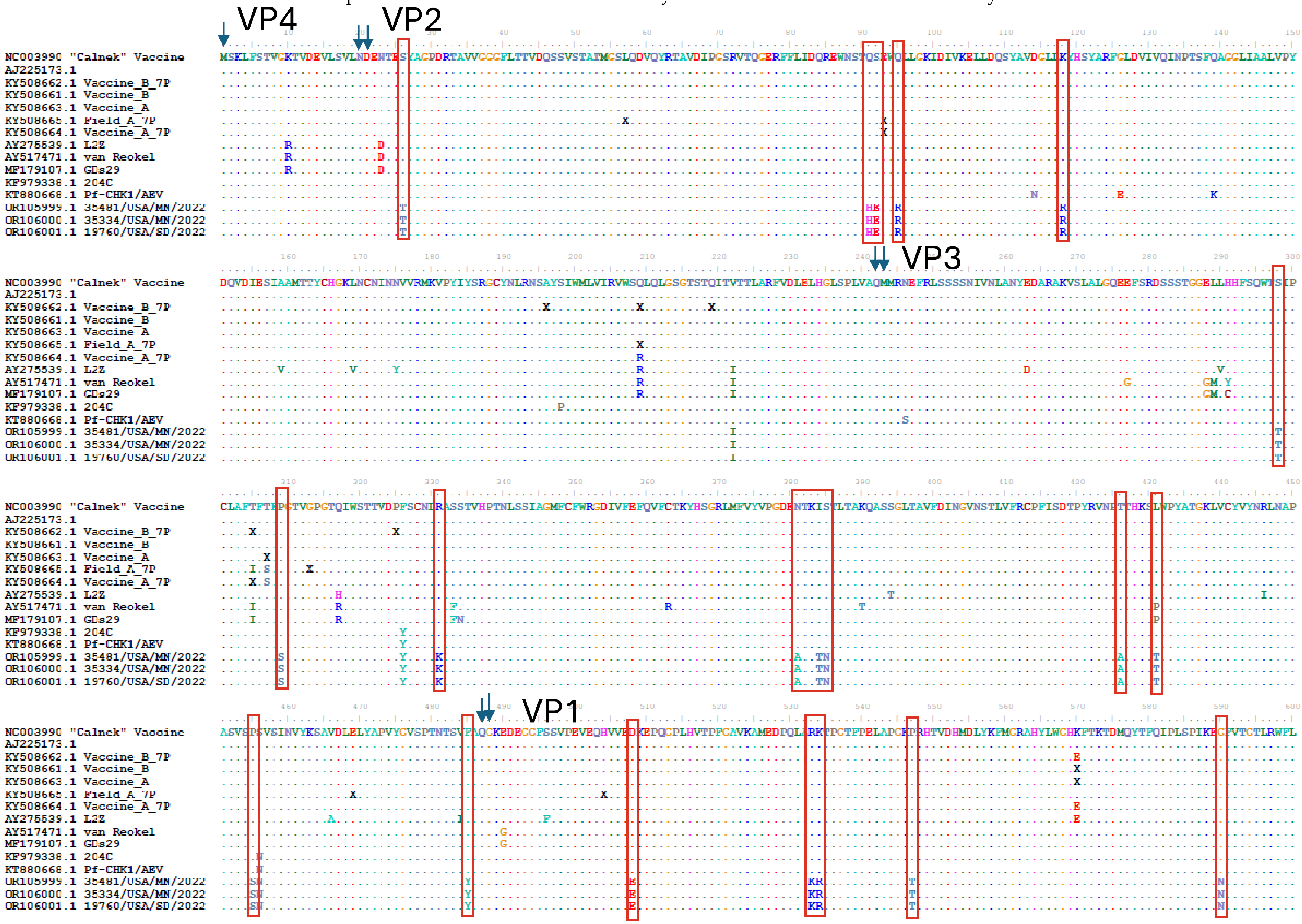

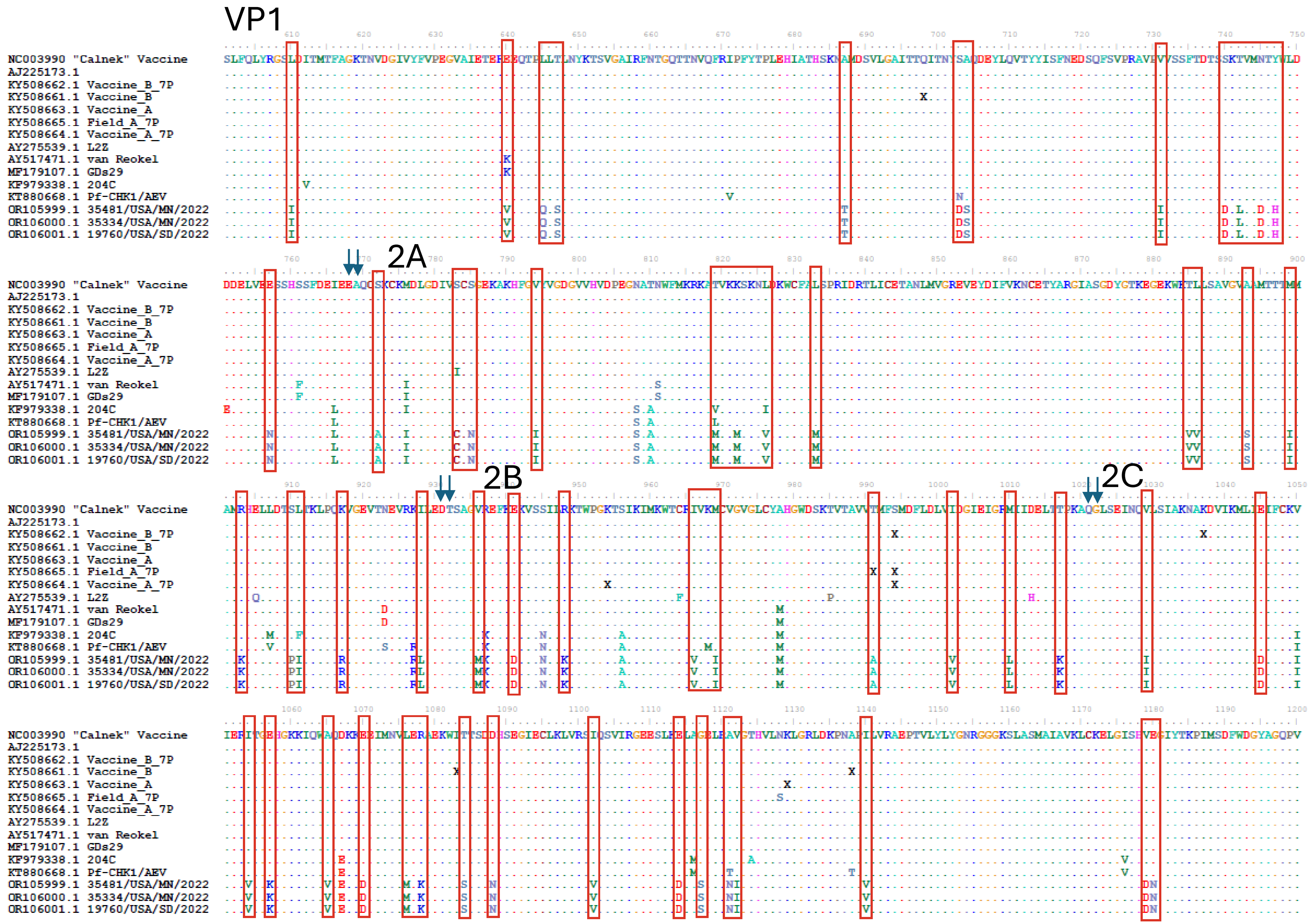
VP1
2A
2B
2C
NC003990 "Calnek" Vaccine
AJ225173.1
KY508662.1 Vaccine_B_7P
KY508661.1 Vaccine_B
KY508663.1 Vaccine_A
KY508665.1 Field_A_7P
KY508664.1 Vaccine_A_7P
AY275539.1 L2Z
AY517471.1 van Reokel
MF179107.1 GDs29
KF979338.1 204C
KT880668.1 Pf-CHK1/AEV
OR105999.1 35481/USA/MN/2022
OR106000.1 35334/USA/MN/2022
OR106001.1 19760/USA/SD/2022

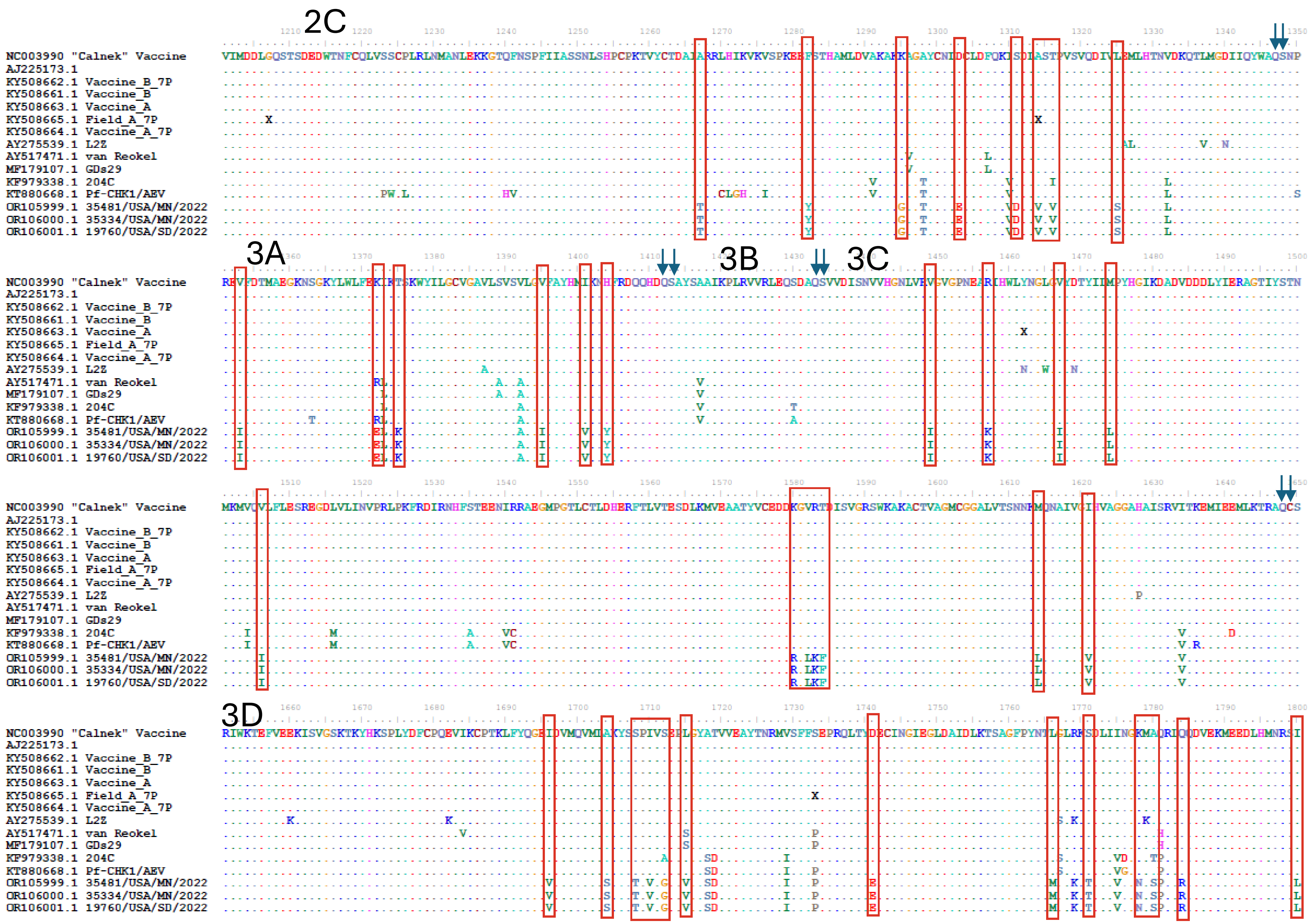
2C
3A
3B
3C
3D
NC003990 "Calnek" Vaccine
AJ225173.1
KY508662.1 Vaccine_B_7P
KY508661.1 Vaccine_B
KY508663.1 Vaccine_A
KY508665.1 Field_A_7P
KY508664.1 Vaccine_A_7P
AY275539.1 L2Z
AY517471.1 van Reokel
MF179107.1 GDs29
KF979338.1 204C
KT880668.1 Pf-CHK1/AEV
OR105999.1 35481/USA/MN/2022
OR106000.1 35334/USA/MN/2022
OR106001.1 19760/USA/SD/2022

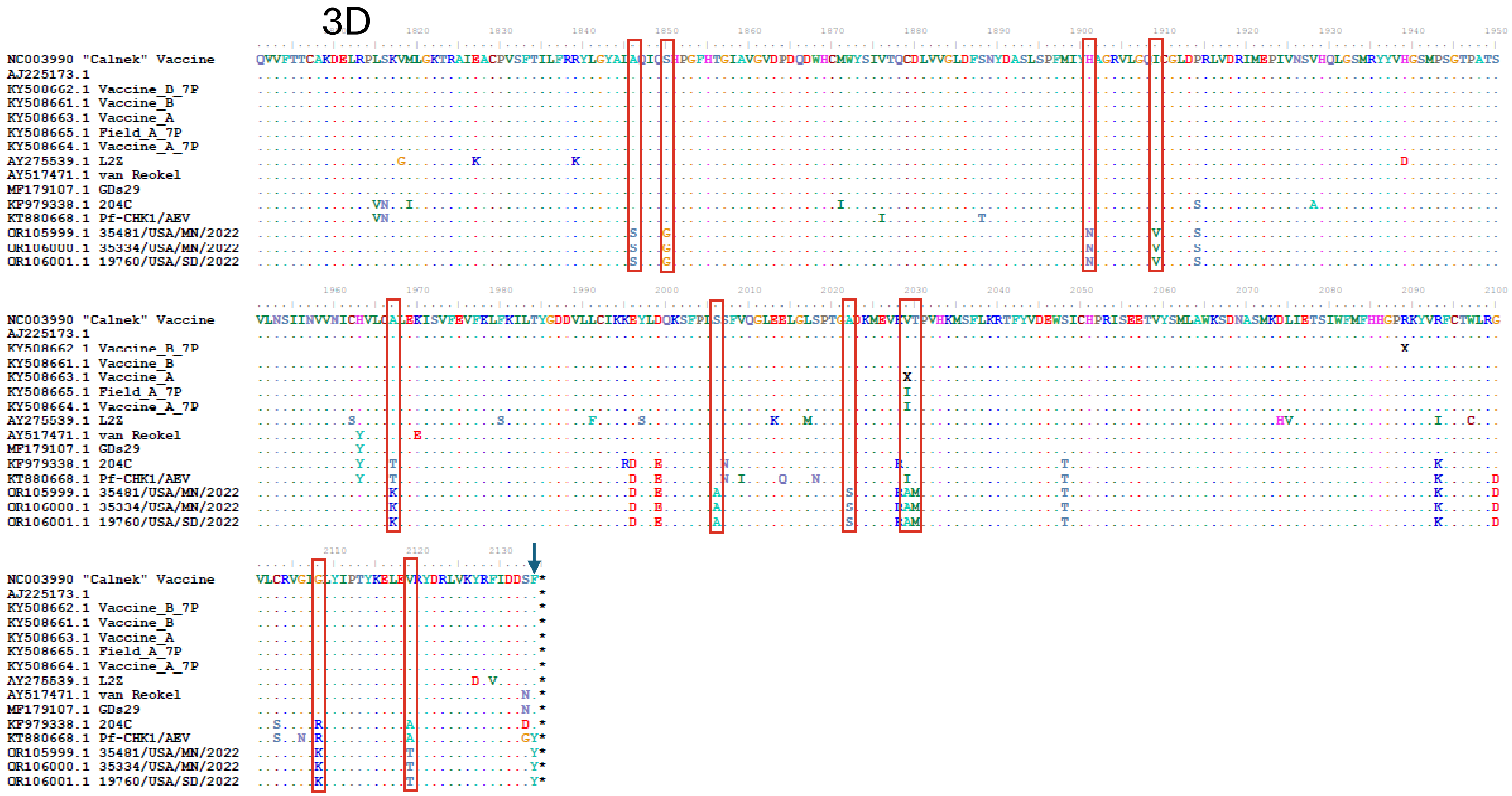
3D
NC003990 "Calnek" Vaccine
AJ225173.1
KY508662.1 Vaccine_B_7P
KY508661.1 Vaccine_B
KY508663.1 Vaccine_A
KY508665.1 Field_A_7P
KY508664.1 Vaccine_A_7P
AY275539.1 L2Z
AY517471.1 van Reokel
MF179107.1 GDs29
KF979338.1 204C
KT880668.1 Pf-CHK1/AEV
OR105999.1 35481/USA/MN/2022
OR106000.1 35334/USA/MN/2022
OR106001.1 19760/USA/SD/2022
